# Unsuccessful attempt at gene-editing by homologous recombination in the zebrafish germ line using the approach of “Rong and Golic”

**DOI:** 10.1007/s11248-012-9607-1

**Published:** 2012-03-21

**Authors:** Rosalind Brookfield, Felix Dafhnis-Calas, Zhengyao Xu, William Brown

**Affiliations:** School of Biology, Queens Medical Centre, Nottingham, NG2 5AN UK

**Keywords:** Gene targeting, Zebrafish, Site-specific recombinase, Transgenesis, Fish

## Abstract

We have investigated the practicality of implementing a strategy for site-specific editing by homologous recombination in zebrafish analogous to that developed by Rong and Golic (Rong and Golic in Genetics 157:1307–1312, 2001) in *Drosophila melanogaster*. We analysed approximately 7,300 offspring from 22 crosses and demonstrated successful excision of the gene editing construct but failed to detect either gene editing or the random integration of the intact editing construct subsequent to excision. The clustering of events in our data set demonstrates that the excision events are not occurring independently and emphasise that a promoter driving high level, tissue-specific transcription in meiotic cells is likely to be necessary if this general approach to site-specific editing by homologous recombination is to fulfil its potential.

**Electronic supplementary material:**

The online version of this article (doi:10.1007/s11248-012-9607-1) contains supplementary material, which is available to authorized users.

## Introduction

Zebrafish are widely used experimentally as models for human development and disease. There is thus a need to investigate the function of individual genes in specific tissues in this organism. The only systematic way in which to carry out such investigations reliably is to generate mutant alleles which can be regulated in a tissue-specific manner. Such alleles can only be generated predictably by the use of site-specific editing by homologous recombination as is routine in the mouse for example. Gene editing by homologous recombination of the mouse germ line is practical because germ line competent embryonic stem cells can be readily derived from certain strains of mice and then cultured and manipulated in the laboratory (Bradley et al. 1984). Such embryonic stem cells have not been derived from zebrafish and so modification of the zebrafish germ line using an analogous approach is currently impossible. There are two potential ways to overcome this technical problem. The first would be to use zinc-finger nucleases to introduce double-strand breaks into the zebrafish germ line genome and thereby trigger gene editing. A double strand break will, in general, be repaired either by non-homologous end joining, leading to a deletion, or by gene conversion from homologous DNA. In human (Porteus and Baltimore 2003) and plant (Shukla et al. 2009; Townsend et al. 2009) cells double strand breaks introduced by zinc-finger nucleases have been used to trigger homologous recombination between the broken gene and a template plasmid. In some cell types this works very efficiently. In human cells for example the target locus will recombine homologously with the exogenous DNA in between 10 and 30 % of the cells containing the broken DNA (Maeder et al. 2008). Zinc-finger nucleases have been used to disrupt genes in zebrafish by two groups (Doyon et al. 2008; Meng et al. 2008). Both described the use of injection of mRNA encoding zinc-finger nucleases into fertilized eggs to generate targeted disruptions of a variety of genes and to generate constitutive loss of function alleles. However the use of zinc-finger nucleases to trigger gene homologous recombination and editing with an exogenous template in zebrafish has not been reported. The overall conclusion that we draw from this work is that while the zinc-finger nuclease approach was the obvious one to take it has, as yet, failed to enable gene editing in zebrafish.

A second potential approach to establishing a methodology for site-specific editing by homologous recombination in zebrafish is suggested by the work of Rong and Golic (Rong and Golic 2000) in *Drosophila melanogaster*. In their experiments the DNA used to template the editing process was interrupted by an I-SceI site and integrated into the germ line genome using a transposon. The integrated template construct was flanked by sites for a site specific recombinase, *Flp* and was then sequentially excised from the germ line genome by conditional expression of *Flp*, and linearized with the meganuclease I-SceI. Rong and Golic remarked in their original publication that their approach was a general one limited only by the ability to construct germ line transgenesis. This may not be true. The editing efficiency originally reported by Rong and Golic and, subsequently, by others is low at about 2 × 10^−3^ per gamete and thus the approach is only practical in organisms which produce large numbers of progeny. Furthermore the need to use breeding to engineer animals with both a transgene encoding an editing construct and a transgene that expresses both I-SceI and a site-specific recombinase means that the approach is also only practical in organisms with short generation times. In zebrafish transgenesis using the Tol2 transposon based system is routine and extremely efficient (Kawakami et al. 2004). Zebrafish have a generation time of about 4 months and a single male can produce thousands of offspring. Thus the approach of Rong and Golic to gene editing would seem to be applicable to zebrafish. Moreover the technique is attractive compared to the zinc-finger nuclease approach in so far as it needs no specialized components but only the reagents and techniques that are routine amongst those working with zebrafish; the ability to engineer DNA and to construct transgenic animals.

We have addressed the problem of establishing gene editing by homologous recombination in zebrafish. We have modelled our approach closely on that of Rong and Golic but not achieved gene editing. Our results suggest that a promoter driving tissue-specific high transcription in meiotic cells would increase the likelihood of success in this type of experiment. This conclusion is applicable to all organisms where there is a need to develop a gene editing by homologous recombination strategy.

## Materials and methods

### Fish manipulation

Single cell *Golb* ^*1*^ */Golb* ^*1*^ embryos were microinjected with approximately 12 pg of both DNA and Tol2 transposase mRNA at a concentration of 100 ng/μl of each per embryo using a Picospritzer II microinjector (Parker instrumentation) a micromanipulator (World Precision Instruments), and a light box (Zeiss KL1500 LCD) under a Zeiss Stemi 2000 dissecting microscope. Surviving fish were raised to sexual maturity and crossed with *Golb* ^*1*^ */Golb* ^*1*^ adults to identify germ line chimeras. The genotype of progeny embryos of F0 Tol2I-SceIscpϕC31-integrase Tol2 microinjected fish was determined by PCR of pooled embryo genomic DNA. Fish transmitting the transgene were crossed to *Golb1* animals and the resulting offspring grown to adulthood. Genotyping was repeated on fin clips of adults. To take fin clips, adult fish were first anaesthetised using Tricaine (3-amino benzoic acid ethyl ester) at 4.2 % strength of 4 g/l stock. Anaesthetised fish were netted out and the caudal fin was amputated using dissection scissors and frozen. In order to extract DNA amputated fins were placed in 100 μl of fin clip lysis buffer (10 mM Tris.HCl pH 8.0, 100 mM EDTA pH 8.0, 0.5 % (w/v) SDS, Proteinase K 25 μg/ml) and incubated at 55°C for 4 h then 95°C for 10 min. Phenol/chloroform extraction was carried out four times and samples were precipitated with 100 % ethanol. Extracted DNA was eluted in 50 μl TE and 1 μl used for PCR analysis (primer sequences are described in the supplementary data). F1 adult fish carrying the transgene were used in experimental crosses. Germ line transgenic Tol2-Ef1α-*attB*-CSKAeGFP2-9-*attP*-RFP-Tol2 (Editing construct) F0 fish were identified by fluorescent microscopy of F1 embryos after outcross to wild-type fish. As would have been expected the yield of germ line transgenic fish generated by the micro-injection of the large 22 kb editing construct was lower than for the smaller 4.6 kb Tol2 I-SceI ϕC31 integrase Tol2 construct. Only 14 % of fluorescent micro-injected transmitted the transgene and on average only 5 % of the progeny of these animals were themselves transgenic. In comparison 45 % of animals micro-injected with a construct with a 4.6 kb insert were transgenic and 25 % of their offspring were transgenic. As a result of the inefficient transgenesis we only obtained two lines that stably transmitted the editing construct. Both of these transmitted the construct to about 50 % of their progeny consistent with a single site of integration. We examined one of the transgenic lines for the integrity of the editing construct by using long range PCR in which one primer was anchored in construct specific DNA. These results were consistent with an intact editing construct except within the region 5′ of exon 2 on the short arm. Further analysis showed that this region contained a long tract of tandemly repeated DNA that had been deleted during the construction of the editing DNA. Although this deletion would be expected to reduce the editing efficiency it is sufficiently far from the site of linearization for the effect to be of minor importance. Transmitting fish were again crossed with *Golb* ^*1*^ */Golb* ^*1*^ and GFP positive fish grown to adult. F1 GFP positive fish were used in experimental crosses. For both lines each F1 founder was from a single F0 germline chimera and each transgenic line was from a single F1 founder. Stocks were maintained by genotyping as above. The data from the crosses carried out in this paper were taken from F1, F2, F3 and F4 fish.

Fish carrying both the Tol2 Ef1α-attB-CSKAeGFP2-9attPRFPTol2 editing and Tol2HSP70 I-SceIscpϕC31 integrase Tol2 constructs were heat shocked as adults to induce expression of I-SceI and ϕC31 integrase. In order to do this adult fish were netted into a small 1 litre tank and transferred into a Techne Hybridiser HB-1D incubator. The incubator temperature was set to 37°C after placing the fish inside. This allowed a gradual increase in water temperature to prevent undue stress. Fish remained in the incubator for 12 h. After heat shock, the incubator was switched off and the water was allowed to return to 28°C before the fish were returned to the routine tanks. Embryos obtained within a week of heat-shocking invariably died soon after hatching but productive mating occurred from a week to several months after the heat shock. The individual pairs were multiply mated. The work was reviewed and passed by the Queens Medical Centre Medical School Ethics Committee, and subsequently approved by the UK Home Office. Project licence (2nd Feb 2006- 2nd Feb 2011): number 40/2893.

### Plasmids and construction

**Fig. 1:**
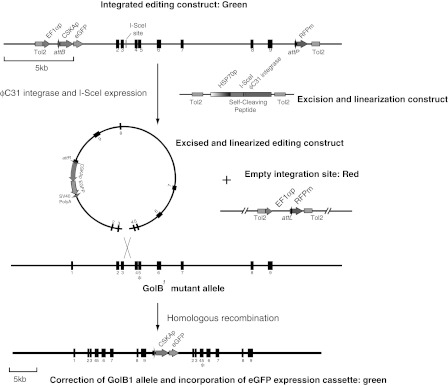
Design of editing experiment. We constructed two lines of transgenic fish using the Tol2 transposon system. The first contained the editing DNA which was isogenic with the *GolB* ^*1*^ allele of the slc24a5 locus and which was interrupted by a site for the I-SceI nuclease. In addition this construct included a constitutively expressed eGFP gene driven by a cyto-skeletal actin promoter (CSKAp). The eGFP gene and editing DNA segments were flanked in turn by attachment sites (*attB* and *attP*) for the ϕC31 integrase and on one side a constitutively active EF1α promoter and on the other a promoterless gene for a membrane bound version of the red fluorescent protein (RFP_m_) (Carreira-Barbosa et al. 2009). Action of the ϕC31 integrase excised the editing DNA and the eGFP gene from the construct and placed the RFP gene under the control of the EF1α promoter (EF1αp). The excised DNA would be predicted to be linearized in the region of homology with the slc24a5 locus as result of the action of the I-SceI nuclease. It would then be able to integrate into the genome either homologously at the slc24a5 locus, non-homologously as a result of DNA repair processes or be lost from the nucleus by diffusion. The second transgenic line contained a transgene that conditionally expressed a fusion protein of the I-SceI nuclease and the ϕC31 integrase separated by a sequence encoding the T2A self cleaving peptide from foot and mouse disease virus (Szymczak et al. 2004). Expression of this fusion protein either as a result of heat shock or as a result of the constitutive activity of the heats shock promoter would be predicted to lead to the production of both I-SceI and ϕC31 integrase following cleaving at the self cleaving peptide

Plasmids were constructed by standard techniques. The editing construct was assembled from DNA amplified from *Golb* ^*1*^ */Golb* ^*1*^ DNA extracted from frozen dead fish (Lamason et al. 2005). The mutant residue in exon 5 of the slc24a5 gene was corrected using a primer containing the wild type sequence. Full details are described in the supplementary data. The excision and linearization expression plasmid (Fig. 1) was constructed by PCR and standard cloning techniques using the plasmid encoding ϕC31 integrase tagged at the carboxy terminus with the SV40 large T antigen nuclear localization signal described in (Dafhnis-Calas et al. 2005) and the plasmid pCMV I-SceI 3xnls which was a gift of Maria Jasin. Details of this construction are also described in the supplementary data.

### PCR and genotyping

BioTaq DNA polymerase (Bioline) was used in most instances for genotyping in a 20 μl reaction. A typical PCR reaction using BioTaq DNA polymerase consisted of template DNA, 200μM dNTPs, 1 × Taq Buffer (16 mM (NH_4_)_2_SO_4_, 67 mM Tris–Cl pH 8.8, 0.01 % Tween-20), 2 mM MgCl_2_, 200nM F primer, 200nM R primer, 2.5 Units of Taq polymerase. The conditions for the PCRs across the *attB/P/L* and *attR* sites are as follows. 40 cycles: 94°C pre-heat step for 5 min, 94°C denaturation step for 10 s, annealing step at 55°C for 15 s, extension step at 72°C for 20 s and a final elongation step at 72°C for 5 min. The primers used in these experiments are listed in Table 2 of the supplementary data.

## Results

### Experimental design

The design of our experiment is set out in Fig. 1. We chose to try to use sequence editing to correct the *GolB* ^*1*^ mutant allele of the slc24a5 gene. This mutant is a C to A nucleotide transversion that converts the codon encoding Tyr^208^ in exon five to a stop codon. Our approach was based upon that of Rong and Golic with modifications that reflect our own experience, recent technical advances and the experimental strengths of the zebrafish system; in particular the utility of fluorescent proteins as markers for screening the nearly transparent fish embryos. In general fluorescent proteins rather than balancer chromosomes were used to follow transgenes in the crosses.

We chose to use an insertion or ends-in approach to editing because this approach was successful in the original *Drosophila* experiments and because such constructs target more efficiently than the more convenient replacement or ends-out constructs (Hasty et al. 1991). In mouse ES cells the presence of strain specific mismatches significantly reduce the frequency of homologous recombination between exogenous DNA and a target gene (te Riele et al. 1992) and so we prepared our editing construct from *GolB* ^*1*^ mutant DNA and corrected the mutant residue by PCR thereby creating a sequence that was identical to the *GolB* ^*1*^ mutant at all corresponding residues except the mutation. (details of the construction are in the supplementary data to this paper). Sequencing confirmed the accuracy of the construction. The editing DNA lacked the promoter and first exon of the slc24a5 gene and was itself incapable of complementing the *GolB* ^*1*^ allele. However we needed to confirm that the region of the slc24a5 gene contained within editing construct was nevertheless capable of correcting the *GolB* ^*1*^ mutant. We therefore assembled a full length derivative of the editing construct including exon 1 and driven by an EF1α promoter (Kawakami et al. 2004) and demonstrated that this complemented the *GolB* ^*1*^ mutant (supplementary data Figure 2). In order to enable linearization of the editing DNA we placed a site for the meganuclease I-SceI between exons three and four. An in vitro experiment (supplementary data Figure 3) showed that this site could be cleaved with I-SceI. An inevitable feature of our approach was that homologous integration of the construct at the slc24a5 locus would not necessarily correct the mutant because any mismatch generated during the homologous recombination reaction could be corrected in favour of the mutant residue. In general mismatches generated as a result of homologous recombination at meiosis are repaired in favour of the resident rather than the invading strand (Orr-Weaver et al. 1988) and if this were true in our experiment we would lack a reliable phenotypic assay for editing. Editing of an insertion construct is usually easy to detect because it generates a duplication of the homologous DNA in the editing construct. Our construct includes approximately 14.8 kb of homology and thus PCR between a duplicated sequence is impractical as a routine screen. We therefore deleted a stretch of 172 residues around the I-SceI site between exons 3 and 4 which allowed us to assay gene conversion by PCR across the short arm of the construct using primers specific for the eGFP gene and for the deleted DNA. This deletion also allowed the slc24a5 DNA within the editing construct to be specifically detected prior to excision using PCR across that region of the third intron that included the deletion. Accompanying the editing DNA was a gene that produced constitutive expression of enhanced green fluorescent protein. This gene was driven by the cyto-skeletal actin (CSKA) promoter (Higashijima et al. 1997). This gene allows us to follow the editing construct both before and after excision on the basis of green fluorescence. Rong and Golic used *Flp* recombinase to excise their editing constructs from the germ line genome. *Flp* recombinase is similar mechanistically to the *Cre* recombinase which is toxic (Loonstra et al. 2001) in mouse cells. Although *Cre* is routinely used in both mouse and zebrafish (see (Blackburn and Langenau 2010) for example) without any evidence of toxicity it is usually expressed for short durations and usually in somatic cells and moreover without any particular attention to the possibility of low level toxicity. Given the scale of our project we decided to therefore chose to use the ϕC31 integrase (Hu et al. 2011; Lister 2011; Lu et al. 2011) as the excision recombinase. There is some evidence that ϕC31 integrase (Ehrhardt et al. 2006) is also toxic however our observations in tissue culture (WRAB, unpublished) indicate that it is less so than the *Cre* recombinase. We therefore flanked the editing DNA ~ CSKAp eGFP segment with attachment sites, *attB* and *attP* for the ϕC31 integrase. We wanted to be able to monitor excision and so we flanked the attachment sites in turn with a constitutive promoter from the EF1α gene and a gene encoding a red fluorescent protein variant that was processed post-translationally into a lipid bound form (Carreira-Barbosa et al. 2009). Excision of the editing DNA CSKA eGFP segment by the ϕC31 integrase would place the RFP coding region close to the EF1α promoter and separated by just a ϕC31 integrase *attL* site. Transient experiments (not shown but see below) demonstrated that this gene was functional and thus red fluorescence acted as a specific marker for successful excision of the editing DNA CSKAp eGFP segment. We cloned this whole segment into a Tol2 transposon vector isolated on a low copy number plasmid pK184, and used it to establish two lines of fish that were transgenic for the editing construct. The total length of the transgenic DNA was 21 kb and this gave lower levels of transgenesis and transmission upon injection construct than a similar construct with a 4.6 kb insert. The differences were manifest in the initial extent of transgenesis, the proportion of transmitting fish and in the proportion of offspring of these fish that in turn were transgenic. The overall reduction in efficiency amounted to about a ten-fold reduction in yield of stably transgenic animals.

The second component of the system was a plasmid that encoded the proteins used to excise and linearize the editing construct. Expression of these proteins needed to be inducible. In zebrafish, as in *Drosophila,* the heat shock protein 70 promoter gives efficient and reproducible induction with a low background. We therefore followed the *Drosophila* example and used the zebrafish HSP70 (Blechinger et al. 2002) promoter to drive expression of the proteins intended to excise and linearize the editing DNA. In order to facilitate the design of the excision and linearization proteins we expressed these as a pro-protein which included the I-SceI meganuclease and the ϕC31 integrase separated by a picorna virus self cleaving peptide (Fig. 1 top). Transient experiments showed that this construct generated both ϕC31 integrase (not shown) and I-SceI activity (see below). We analysed the integrity of the editing construct in the transgenic fish by long range PCR using primers that specifically annealed either to the flanking DNA encoding the fluorescent proteins or the I-SceI site and to DNA within the targeting construct. The results of these analyses were consistent with the integrity of the editing construct except for a tandemly repeated sequence 5′ of exon 2 which subsequent analysis showed had been deleted during the construction. This deletion was unlikely to compromise editing significantly because it was more than 1 kb from the I-SceI site and so we continued to use these fish. Both transgenes always segregated as single Mendelian loci in the crosses discussed below and were thus were present at single loci. We cannot exclude the possibility that they are present in multiple copies but this seems unlikely given the mechanism of transposon integration and the low frequency of transgenesis that we observed with these large transgenes.

### A double strand break generated using I-SceI meganuclease promotes intra-molecular gene conversion in zebrafish embryos

**Fig. 2:**
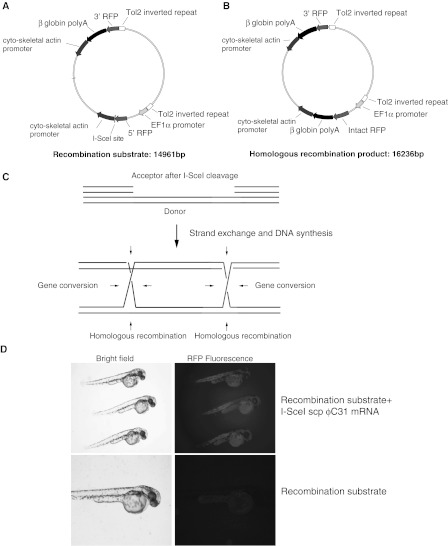
Homologous recombination activity is readily detectable in zebrafish embryos. A plasmid termed recombination substrate (**a**) was prepared by routine recombinant DNA techniques (sequence present in supplementary information). This plasmid contained two incomplete segments of a gene encoding red fluorescent protein (RFP). One of these segments was flanked on one side by an I-SceI site and functioned as acceptor for sequence information contained within the other. Homologous recombination or gene conversion triggered by cleavage at the I-SceI site reconstitutes the functional RFP gene (**b**, **c**) as result of strand exchange and DNA synthesis and confer red fluorescence upon the injected embryos. **d** Zebrafish eggs co-injected with the recombination substrate plasmid and mRNA encoding the ϕC31 integrase scp I-SceI fusion protein specifically showed red fluorescence

We needed to confirm that the I-SceI meganuclease encoded in the ϕC31 integrase scp I-SceI fusion protein was active and could trigger homologous recombination in zebrafish. Plasmids actively recombine homologously in mammalian cells (Folger et al. 1985) when present extra-chromosomally and so we decided to assay I-SceI by its ability to trigger intra-molecular extra-chromosomal homologous recombination in zebrafish embryos. We are unaware of any work showing that zebrafish are capable of homologous recombination and thus it also seemed useful to demonstrate that that they did not differ in this respect from mouse cells for example. We therefore prepared a recombination substrate plasmid containing two overlapping segments of a gene encoding red fluorescent protein (Fig. 2a). The 3′ segment and polyadenylation signal functioned as the sequence donor and the 5′ segment and promoter functioned as the acceptor. These two segments of the RFP overlapped by 417 bp. Downstream of both the donor and acceptor cassettes was 1,168 bp of the cytoskeletal actin promoter which functioned solely as a region of homology. An I-SceI site was placed between the 5′ segment of the RFP gene and the cytoskeletal actin promoter. Homologous recombination between these segments triggered by I-SceI would reconstitute a functional RFP gene (Fig. 2b) as a result of the homologous recombination reaction shown in Fig. 2c. Co-injection of the recombination substrate plasmid and mRNA encoding the ϕC31 integrase scp I-SceI fusion protein led to the appearance of red fluorescence in the injected embryos which was not detectable if the mRNA encoding the fusion protein was not present in the injected material (Fig. 2d). In total 185 embryos were injected with a mixture of both the recombination substrate and the mRNA encoding the ϕC31 integrase scp I-SceI fusion protein. We recovered 89 viable embryos of which 73 showed red fluorescence. Approximately 50 % survival of embryos survived was typically observed in control embryo manipulations and is not specific to the experiment. The failure of 10–20 % of embryos to show any fluorescence is also typical of control experiments and arises either from muddling embryos in the injection dish or from mis-injection. 54 embryos were injected with the recombination substrate alone, 24 were recovered alive and none showed any red fluorescence. An extra-chromosomal recombination assay of this type however does not give any information about the rate of germ line gene editing of the sort we attempt below.

### Conditional excision and integration of damaged editing construct DNA into the fish genome

**Fig. 3:**
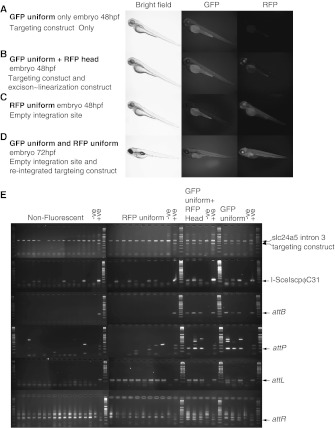
Analysis of the embryos generated in the experiments described in this paper. **a**–**d** In the course of this experiment we generated four types of fluorescent embryos and fish. All were *GolB* ^*1*^ */GolB* ^*1*^ homozygotes and hypo-pigmented. **a** The first contained the integrated editing construct and showed uniformly green fluorescence. **b** The second contained the integrated editing construct and the excision~linearization construct which in some fish is active in the brain and eyes. Such fish showed green fluorescence uniformly with red fluorescence in the head and eyes. This could be explained by expression of the RFP gene activated by site specific recombination mediated by the ϕC31 integrase between the *attB* and *attP* sites in the integrated editing construct. Such fish should contain an *attL* site. **c** The third showed uniform red fluorescence and were the progeny of the type of fish shown in (b). These fish could be explained as containing the empty integration site (see Fig. 1 for details) and should contain an *attL* site (**d)**. The fourth type of fish showed uniform green and uniform red fluorescence and could be explained by their containing both an empty integration site and a editing construct that had re-integrated in the genome at a position other than the slc24a5 locus or had recombined homologously with the slc24a5 but had failed to correct the *GolB* ^*1*^ allele. Such fish should contain an *attL* site and an *attR* site. **e** Analysis of the fish illustrated in (**a**-**c)** by PCR for the presence of the editing construct, the excision linearization construct and the ϕC31 integrase attachment sites confirmed the interpretations of the fluorescence phenotypes and in addition demonstrated that the ϕC31 integrase scp I-SceI fusion protein could be present in the fish without detectable activity in the head or eyes. In this cross only three fish that were uniformly fluorescent green were generated and all of these were also transgenic for the ϕC31 integrase scp I-SceI fusion construct. The *attR* PCR included significant background. This reflects the fact that the template is present in only a small proportion of cells in the respective embryos and consequently the PCR was continued for 45 cycles but the site was not detectable in these embryos. The presence of the *attL* sites in the uniformly green fluorescent embryos presumably reflects a low level of ϕC31 integrase scp I-SceI in these animals despite the absence of high level expression in the head and eyes. The weakly penetrant phenotype associated with the ectopic expression of the ϕC31 integrase scp I-SceI fusion was also evident in the breeding experiments. (see the supplementary data for a discussion) DNA size markers (Q4 Bioline) flanked the individual analyses

In order to assay the feasibility of using conditional excision of a germ line integrated editing construct as the basis of gene editing in zebrafish we crossed fish transgenic for the excision~linearization expression construct with fish transgenic for the editing construct. This produced two sorts of fluorescent fish. The first was similar to the parent with the transgenic editing construct and was uniformly fluorescent green (Fig. 3a). The second type showed uniform green fluorescence and red fluorescence in the head (Fig. 3b). The heat shock promoter is known to show activity in the brain and eyes independently of heat (Blechinger et al. 2002) and so we concluded that this second type of fish contained both the excision linearization construct and the editing construct. These were the fish which we wanted to use for our gene editing experiment and so we used them in a second set of mating experiments. In these mating experiments we crossed the fish that were uniformly green and red headed with *GolB* ^*1*^ */GolB* ^*1*^ fish. We did this both before and after heat-shocking of the uniformly green and red headed fish. Individual crosses were multiply mated and in some cases produced thousands of embryos (Supplementary data; Table 3). This second type of cross generated two more types of fluorescent fish in addition to the fish that were both uniformly green and red headed and the uniformly green fish that we had identified earlier; the first showed uniform red fluorescence (Fig. 3c) and the second, which was found much more rarely, showed both uniform green and uniform red fluorescence (Fig. 3d). The proportion (Table 1) of the uniformly red fish increased after heat shock which together with their appearance suggested that they corresponded to fish with an empty integration site (Fig. 1) that had been generated by excision of the editing construct from the genome by the action of the activity of the ϕC31 integrase. The uniformly green and uniformly red fish may correspond to fish which were predicted to contain both an empty integration site and a editing construct ~eGFP cassette which had re-integrated into the genome after excision mediated by the ϕC31 integrase. We analysed non-fluorescent fish, uniformly green fluorescent fish, uniformly red fluorescent fish and the uniformly green fluorescent fish with a red head by PCR for the presence of the editing construct, the ϕC31 integrase scp I-SceI fusion gene and for the *attB, attP, attL and attR* sites for the ϕC31 integrase. The results (Fig. 3e) were consistent with the phenotypes except that the three exclusively green fluorescent fish we analysed contained the ϕC31 integrase scp I-SceI fusion gene and contained detectable *attL* sites. We conclude that in these fish the fusion gene is active but at too low a level to yield a red head. This conclusion was consistent with the results of the segregation analysis (not shown) which also showed weak penetrance of the red headed phenotype associated with the ϕC31 integrase scp I-SceI fusion gene.

**Table 1:** Phenotypes of fish generated in crosses between *GolB* ^*1*^/*GolB* ^*1*^ fish and fish transgenic for both the excision-linearization construct and the integrated editing construct

Fluorescent phenotype	Number before heat-shock ( %)	Number after heat-shock ( %)
No fluorescence	571 (43 %)	3,314 (45 %)
Uniform green only	443 (34 %)	1,638 (22 %)
Uniform red only	80 (6 %)	1,661 (23 %)
Uniform green and red head fluorescence in head	210 (16 %)	677 (9 %)
Uniform green and uniform red red	0	28 (0.4 %)
Efficiency of excision	12 %	46 %

The numbers in this table summarize the data contained within Table 3 of the supplementary data. The efficiency of excision was calculated on the basis that there is only one copy of the integrated editing construct is present in the cross. The fish described in this table resulted from 22 pairs of fish (crosses) each of which was mated several times over a period of about a year

The most interesting fish generated in the crosses after heat-shock were those which showed both uniform green and red fluorescence because these were candidates for fish which contained both an empty integration site and re-integrated editing construct (Fig. 1). We carried out 22 crosses after heat-shocking (supplementary data Table 3) of which only four such crosses yielded progeny which were both uniformly green and red fluorescent. These progeny were generated in multiple individual matings over the course of many months. In these four crosses 2, 3, 7 and 16 progeny were obtained which expressed both the red and green fluorescent proteins uniformly (see supplementary data Table 3 for the entire data set). This clustering of candidate re-integration events suggests that they were not occurring independently. Calculation of the deviation from independence confirmed that this was significant at the 0.1 % level (JFY Brookfield, personal communication). These data therefore indicate that the excision and possible re-integration reactions are occurring in the precursors of meiotic cells and that these cells have divided many times before meiosis. Thus although we have analysed more than 7,000 embryos generated in 22 crosses we have probably generated only four candidate independent candidate re-integration events. We analysed fourteen embryos from the crosses which yielded 7 and 16 progeny containing candidate re-integration events by PCR. These offspring were either from the male SN510 or from the male SN631. The offspring of the male SN510 were all *attB* ^−^ *, attP* ^−^, *attL* ^+^ *;* as one would expect from the uniform red fluorescence but they were also *attR* ^−^. This was unexpected given that they were GFP positive and so we concluded that the excised editing construct had been degraded before integrating into the genome of these fish. Thus these fish were not informative for editing. The offspring of the male SN631 were all *attB* ^+^ *, attP* ^+^ *, attL* ^+^ *, attR* ^+^ but also contained the ϕC31 integrase scp I-SceI fusion gene. The presence of both *attB, attP* sites, diagnostic of the integrated editing construct, and of the *attL* site diagnostic of the empty integration site suggested that the construct had been duplicated in the germ line prior to the excision reaction. The *attL* ^+^ *, attR* ^+^ could then be explained by the the ϕC31 integrase scp I-SceI fusion gene which was also present in the these fish. Thus these fish also provided no evidence either for the editing reaction planned in Fig. 1 or for the more likely excision and random integration of the editing construct. Although these fish were not simple re-integrants they do indicate that re-integration of an excised targeting construct, albeit damaged, does occur at detectable frequencies. This interpretation is however based upon indirect data and will need to be confirmed by more molecular analysis in a system that gives many more independent excision events. It may also seem to be at variance with the low molecular efficiency of transgenesis seen in the initial egg injections and thus some discussion of the quantitative aspects of this interpretation are justified. In the initial egg injections we injected 12 pg of a 25 kb DNA molecule which is equivalent to 600,000 molecules and only detected transgenesis at an efficiency of a few percent. The fertilized egg is a hemisphere with radius of 150 μm. We do not know the radius of a zebrafish germ cell nucleus but let us assume that it is similar to that of a HeLa cell and is approximately 3 μm. Making the simplest assumption that the injected DNA can efficiently equilibrate with the nuclear contents, the DNA injected into the egg is therefore present in the nucleus at a concentration which is only four times greater than that that of a single DNA molecule in the nucleus to start with. Given the fact that the chromatin in the fertilized egg is likely to be relatively condensed because it is not being transcribed while any excised DNA is already within the transcriptionally active nucleus there is no discrepancy between the interpretation of the data to suggest that targeting DNA has re-integrated and the low efficiency of initial transgenesis.

## Discussion

Gene editing by homologous recombination is an indispensable technique both for biotechnology and for the rigorous analysis of gene function. Gene editing is however routine in only a few metazoan organisms and as such it would be valuable to establish an experimentally simple strategy that could be applied generally. The approach taken by Rong and Golic (Rong and Golic 2000) to gene editing in *Drosophila melanogaster* is potentially such a strategy. We have shown however that this approach has limitations and we discuss how these limitations could be overcome in zebrafish. Although our results are negative they are worth reporting because the approach of Rong and Golic is a general one and, it would seem, needs to be developed if progress is to be made in the rigorous analysis of gene function of emerging model organisms.

The main limitation that prevents the simple implementation of the Rong and Golic (Rong and Golic 2000) strategy in zebrafish is the fact that that the heat-shock promoter cannot be induced specifically, easily, to high level and exclusively in meiotic cells. Consequently any event that may represent the re-integration of the editing construct does not occur independently amongst the offspring. This was also observed in the *Drosophila* experiments of Rong and Golic but has not been an impediment to the utility of the approach because it is easy to arrange large numbers of *Drosophila* matings. Breeding and arranging fish to mate is however time consuming and our results show that the application to zebrafish of the approach to gene editing using a heat shock promoter would likely require hundreds of thousands of inductions and tens of thousands of mating experiments. This is beyond the capacity of most small laboratories. However were a meiosis specific promoter to be used to drive expression of the gene encoding the excision~linearization protein then all of the re-integration events would have been likely to be independent and one would have had a corresponding increase in the chance of detecting a editing event in any one mating. The use of a meiosis specific promoter to drive chromosome engineering reaction driven by site-specific recombination has been described in the mouse (Herault et al. 1998). In these experiments the promoter of the synaptonemal complex protein 1 SYCP1 protein was used. Genes whose expression is meiosis specific have also been described in zebrafish (Zeng and Gong 2002) and medaka (Lo et al. 2008) and it would seem reasonable to try to test the promoters of each of these genes for their ability to drive meiosis specific expression of the gene encoding the excision~linearization protein. Fundamental however to any estimation of the practicality of the Rong and Golic approach to gene editing in fish is also an estimate of the random re-integration frequency and of the background of non-specific events that we have detected. Given an appropriate promoter the system described here may be able to be adapted to make these measurements in this and other systems.

## Electronic supplementary material

Below is the link to the electronic supplementary material.

Supplementary material 1 (PDF 2175 kb)
