# Supplementary material for: Unsuccessful attempt at gene-editing by homologous recombination in the zebrafish germ line using the approach of “Rong and Golic”
Source: Transgenic Res. 2012 Mar 21;21(5):1125–36. doi: 10.1007/s11248-012-9607-1 (PMC3432782; doi:10.1007/s11248-012-9607-1)

Brookfield et al Supplementary data

Construction of pKTol2 *attB* CSKApEGFP a*ttP* RFP plasmid

The pT2KXIG plasmid (a kind gift of Dr. Kawakami) was first partially cut with NotI and then blunted by using the polymerisation activity of the Klenow fragment followed by religation of the plasmid in order to delete the NotI site at the 3' end of the TOL2 repeat element. The pT2KXIGΔNotI was then cut with BamHI and ClaI and next it was ligated to a BamHI/ClaI linker containing the *attB* site for the ϕC31 integrase and the restriction sites for NdeI, NotI, EcoRV and BamHI enzymes. The introduction of the linker deleted the ClaI and BamHI restriction sites in the plasmid pT2KXIGΔNotI. The plasmid pT2KXIGΔNotI*attB* was digested with BamHI and next ligated to a BamHI PCR fragment that contained the coding sequence for the membrane bound red fluorescent protein (mRFP). The 5'end primer used in the RFP PCR introduced the *attP* site for the ϕC31 integrase. The plasmid derived from the above ligation was termed Tol2ef1*attBNdeINotIattP* RFP. After that, the XhoI site upstream the eF1α promoter in the Tol2ef1*attBattP* RFP plasmid was deleted by cutting the site with XhoI and blunting the ends using the Klenow polymerase activity. Subsequently, a ClaI linker was cloned into the NdeI site. The cassette containing the Tol2eF1α*attBClaINotI attP* RFP was then amplified by PCR. The primers used in the PCR introduced an XhoI restriction site. The PCR product was cut with XhoI and then ligated with the low copy number plasmid pK184 previously cut with SalI. The new plasmid was named pkTol2ef1*attBattP* RFP. It was digested with ClaI and then ligated to the expression cassette CSKApEGFPployA previously modified with ClaI. The new recombinant molecule derived from that ligation was termed pkTol2ef1*attB* CSKApEGFPployA *attP* RFP.

Construction of the Tol2HSP70IsceIscp*ΦC31* plasmid

The pT2KXIG plasmid (obtained from Dr. Kawakami) was first cut with BamHI and ClaI to delete the EGFP gene and then it was blunted by using the polymerase activity of the Klenow fragment. The blunting reaction of the BamHI/ClaI pT2KXIG plasmid followed by a self-ligation reaction regenerated the BamHI restriction site. The product of the ligation, pT2KXIGΔ EGFP, was digested with the XhoI restriction enzyme followed first by a partial filling reaction and then by complete digestion with the SalI restriction enzyme. The XhoI/PF/SalI pT2KXIGΔ EGFP plasmid did not contain the e1F promoter and it was used as cloning vector of the HSP70 promoter from zebrafish. The fragment containing the heat shock promoter was previously modified with the restriction enzyme BamHI followed first by a partial filling reaction with dGTP and dATP and then by a complete digestion with the restriction enzyme, SalI. The new plasmid, Tol2HSP70, was first cut with the BamHI restriction enzyme and next ligated with a BamHI fragment containing the gene encoding the 3xnlsIsceI endonuclease fused through the T2A self cleave peptide linker to the 3'nlsϕC31 integrase gene which was amplified from a plasmid kindly sent to us by Maria Jasin using the oligonulceotides IsceI Fow: `actgggatccatgggatcaagatcgccaaaaaag` and I-SCEI SCPr `attaggatccagatctagtgggcctgggttctcctccacgtcgccgcaggtcaacaagctgccgcggccctctttcaggaaagtttcgga`. The IsceI endonuclease contained a nuclear localisation signal (nls) at the amino terminus of the protein and the ϕC31 integrase had an nls signal at the carboxy terminus. The new expression plasmid was termed Tol2HSP70IsceIscpϕC31integrase.

Supplementary data: Table 1: primers used to assemble the targeting construct.

1. Primers used for isolate genomic DNA from upstream exon 4 up to downstream exon 7 of GOLB gene.
2. Primers used to amplify genomic DNA from upstream exon 7 to downstream exon 9.
3. Primer used to correct mutation in the exon 5 of the GolB gene. It was utilized with primer Golb3 end F-2.The PCR product amplified region from exon 4 to exon 5.
4. Primers used to correct mutation in exon 5 of the GolB gene. The PCR product contained exon 5 and 6.
5. Primers used to isolate 5'end of the targeted construct. It contained exon 2 and 3 of the GolB gene.
6. Primers used to isolate the Tol2ef1*attBClaINotI attP* RFP cassette.
7. Linker used to introduce the *attB* site for the ϕC31 integrase.

Primers used to amplify the membrane bound RFP and also introduce the *attP* site for the ϕC31integrase.

Supplementary data: Table 2: primers used to analyse fish.

The first three pairs of primers were used to check for sequence targeting at the slc24a5 locus. The results were negative and are not shown. The other primer pairs are those used in the PCR analyses illustrated in figure 3.

Supplementary data: Table 3: outcomes of the crosses carried out in the work described in this paper.

This table enumerates the details summarised in table I of this paper. Fish indicated in bold type in columns one or two are the transgenic fish in the respective crosses.

Supplementary data figure 1: construction of the pKTol2attBattPCSKApEGFP/GolB 2-9 plasmid.

Two long range PCR reactions were performed to isolate exons 4 to 9 from the sLc24a5 gene. The first PCR product (6 kb) contained the exons 4, 6 and 7 and the mutated exon 5. The primers used for this PCR introduced the restriction sites for XhoI and the I-SceI endonuclease site. The second PCR amplified a 6.1kb fragment that contained exons 7, 8 and 9. The primers used in this PCR introduced a NotI restriction site. Both PCR reactions were performed by using the Expand long template PCR system (Roche). The PCR product that contained the exons 4 to 7 was then digested with the restriction enzymes XhoI and Asp718 and the PCR fragment that included exons 7 to 9 was digested with the restriction enzymes Asp718 and NotI. Both modified PCR products were then cloned in a three way ligation into a pBS vector to produce a fragment including exons 4 to 9; pBS GOLB 4-9. Next, a two step PCR reaction was carried out to change the stop codon sequence (TAA) present in the exon 5 for a DNA sequence that specified the aminoacid tyrosine (TAC). First, two individual PCR reactions were used to introduce a base pair change into exon 5. Both PCR fragments were then annealed in a second reaction and amplified with external primers. This PCR product was first digested with XhoI and EcoRI and then ligated into the pBSGOLB4-9 previously cut with XhoI and EcoRI. The new plasmid had the exon 5 with the corrected mutation and it was named pBSGOLB4-9/fixed exon5. A DNA fragment including exons 2 and 3 was then isolated by PCR. The primers used in that reaction introduced the restriction sites for XhoI and a site for the I-SceI endonuclease. The DNA fragment including exons 2 and 3 was digested with XhoI and I- SceI and then ligated with the pBSGOLB4-9/fixed exon5 previously also cut with XhoI and I-SceI. To produce pBS, pBSGOLB2-9. This plasmid was digested with the restriction enzyme NotI and the released fragment including exons 2 to 9 was subsequently ligated into the pkTol2*attB*CSKApEGFP*attP* RFP (construction described in supplementary data) vector previously cut with NotI. The DNA construct derived from the ligation was named pkTol2*attBattP*CSKApEGFP/GolB 2-9 or more commonly “the targeting construct”.

Supplementary data figure 2: a reassembled slc24a5 gene reconstitutes pigmentation in *GolB*^{1} embryos.

The targeting construct shown in figure 1 was modified by the addition of the first exon of the slc24a5 gene and a promoter for the EF1α translation factor (Kawakami et al., 2004), the resulting plasmid termed the promoter construct was injected into *GolB*^{1}/ *GolB*^{1}fish eggs together with mRNA encoding the Tol2 transposase and visualized under phase microscopy 24 hours later . An un-injected *GolB*^{1}/ *GolB*^{1} embryo and a wild type embryo are illustrated as controls for the specificity of the appearance of pigment in the experimental sample.

Supplementary data figure 3: cleavage of the targeting construct with I-SceI. A; Agarose gel resolving digest of targeting construct plasmid with NotI alone or NotI and I-SceI. B map of the targeting construct showing relevant sites and important features.

Reference

Kawakami, K., Takeda, H., Kawakami, N., Kobayashi, M., Matsuda, N. and Mishina, M. A transposon-mediated gene trap approach identifies developmentally regulated genes in zebrafish. *Dev Cell* **7** (2004), pp. 133-44.

Table I supplementary data; Brookfield et al

a	Golb3 end F-2	atgcctcgagtagggataacagggtaataggccacgcacacacaggttgcc	1
b	Asp7 18 Rev2	actgagatgaagcgttcacgggact	
c	Asp7 18 Fow2	agtgctcgagatctgctgctgcttttcctccgtgt	2
d	Golb3EndRev 3	cgatctgcggccgcagatccgccacttttgacaaacaat	
e	correct mut rev	ctcaccagtaaactctgttatctg	3
f	correct mut fow	cagataacagagtttactggtgag	4
g	Exon- 7Rb	tgtcaaaccgagagcagatg	
h	Left arm forward 4	agtctgtcgacgcggccgcaaccagaaaacaggagaggagaaga	5
i	LeftarmRev3 Bam-2	atgcaggatccattaccctgttatccctacccgatagttctgtagtcctcaaca	
J	Tol2 Forward	atcgctcgagacagaggtgtaaaaagtact	6
K	Tol2 Reverse	atcgctcgagaggcaacaaaagctggacct	
L	attBFow BamHI ClaI	gatcaccgcggtgcgggtgccagggtgtgcccttgggctccccagggcacccctccacatatgatatcgcggccgcggatcc	7
G	attBrev BamHI/ ClaI	cgggatccgcggccgcgatatcatatgtggaggggtgccctggggagcccaagggcacaccctggcacccgcaccgcgg	
H	attP/mRFPForward BamHI	tgctggatccgtagtgccccaactggggtaacctttgagttctctcagttgggggcgtag	8
I	RFP Rev	agctggatcctcacaaaatgctacacttcg	

Table 2 supplementary data; Brookfield et al

PCR	FORWARD PRIMER	REVERSE PRIMER
Positive Control TC Check	ATCAGTCATCGGACGTACCCGACAACCACTACCTGAGCACCCA	ATGCATTACCCTGTTATCCCTACCCGATAGTTCTGTAGTCCTCAGGA
Positive Control Genomic Check	AGTCTGTCGACGCGGCCGCAACCAGAAAACAGGAGAGGAGAAGA	TGAGCGTAACACGGTTACCACACTGACGGATCTCTGCACTGA
Positive Control Recombination Check	ATCAGTCATCGGACGTACCCGACAACCACTACCTGAGCACCCA	TGAGCGTAACACGGTTACCACACTGACGGATCTCTGCACTGA
attB	TCATGCCTTCTTCTCTTTCC	CATGTTTTTCCCCTCACTGT
attP	CTTCAGCAGACCTCCTCATT	CCTTGGTCACCTTCAGCTT
attL	GTTGTGCTGTCTCATCATTTGG	AACTCCTTGATGACGTTCTCG
attR	TGATTGTTTGTCAAAAGTGGCGG	GGGTGTGTATTTTCCTAGTGC
RFP	AGATCAAGATGAGGCTGAAGC	ATGGTGTAGTCCTCGTTGTGG
I-SceI site in targeting construct	GTTTCAGGTCTTGGTCTCTC	TATATTTCAGCATCCCTGTATG
I-SceIscpϕC31 excision construct	CCTGAAAGAGGGCCGC	CCGGCGCTTCGCTGAAA

Table 3 supplementary data; Brookfield et al

Parents		Before heat-shock						After heat-shock					
Female	Male	Not fluorescent	GFP	Whole RFP	GFP + RFP head	Whole RFP + GFP	Viable embryos	Not fluorescent	GFP	Whole RFP	GFP + RFP head	Whole RFP + GFP	Viable embryos
**SN510 12x29(A)**	SN445 GolB1	1	4	0	3	0	8	2	5	0	2	0	9
**SN510 12x29 (B)**	SN424 GolB1	2	18	1	5	0	26	80	52	11	31	0	174
SN424 GolB1	**SN510 12x29**	6	19	1	10	0	36	547	277	116	88	16	1044
SN564 GolB1	**SN533 11x29 C**	68	47	2	19	0	136	236	89	58	52	0	435
**SN533 11x29 (A)**	SN564,480 GolB1	2	5	0	0	0	7						
**SN533 11x29 (B)**	SN564,480,445 GolB1	6	11	0	0	0	17						
**SN565 12x29(A)(Fin clip)**	SN480 GolB1	11	10	0	5	0	26	174	161	108	25	2	470
SN564 GolB1	**SN595 12x29 (A)**	14	5	0	2	0	21	134	47	31	12	0	224
SN564 GolB1	**SN595 12x29 (B)**	14	6	0	6	0	26	37	15	15	15	0	82
SN564 GolB1	**SN565 12x29 (A)**	52	25	15	24	0	116	328	105	100	63	0	596
**SN565 12x29 (Bf)**	SN564 GolB1	13	10	4	7	0	34	978	386	784	143	0	2291
SN564 GolB1	**SN565 12x29 (Bm)**	40	29	17	21	0	107	22	12	2	9	0	45
SN564 GolB1	**SN533 11x29 (E)**	13	17	0	6	0	36	53	62	7	12	0	134
SN564 GolB1	**SN632 12x29 (B)**	40	14	8	5	0	67	79	34	36	4	0	153
**SN632 12x29 A**	SN564 GolB1	22	21	0	8	0	51	110	119	95	34	0	358
SN564 GolB1	**SN640 12x29**	40	13	7	14	0	74	9	5	4	3	0	21
**SN631 12x29**	SN564 GolB1	0	0	0	1	0	1						
SN564 GolB1	**SN631 12x29**	15	2	6	1	0	24	153	21	124	10	7	315
**SN632 12x29 B**	SN564 GolB1	6	13	1	7	0	27						
**SN627 12x29**	SN564 GolB1	63	57	12	15	0	147	110	71	119	39	3	342
**SN632 12x29 C**	SN564 GolB1	22	23	3	19	0	67	70	56	30	23	0	179
SN564 GolB1	**SN739 12x29 B**	24	11	0	9	0	44	51	18	1	16	0	86
**SN739 12x29 A**	SN564 GolB1	0	4	0	1	0	5	7	15	5	5	0	32
**SN776 12x29 A**	SN736 GolB1	26	22	2	18	0	68	9	36	0	20	0	65
SN736 GolB1	**SN776 12x29 B**	0	0	0	0	0	0						
SN736 GolB1	**SN776 12x29 C**	9	4	1	4	0	18	47	18	4	28	0	97
SN736 GolB1	**SN776 12x29 D**	0	1	0	0	0	1	78	34	11	43	0	166
SN736 GolB1	**SN776 12x29 E**	62	52	0	0	0	114						
**Totals**		**571**	**443**	**80**	**210**	**0**	**1304**	**3314**	**1638**	**1661**	**677**	**28**	**7318**

Figure 1; supplementary data Brookfield et al.

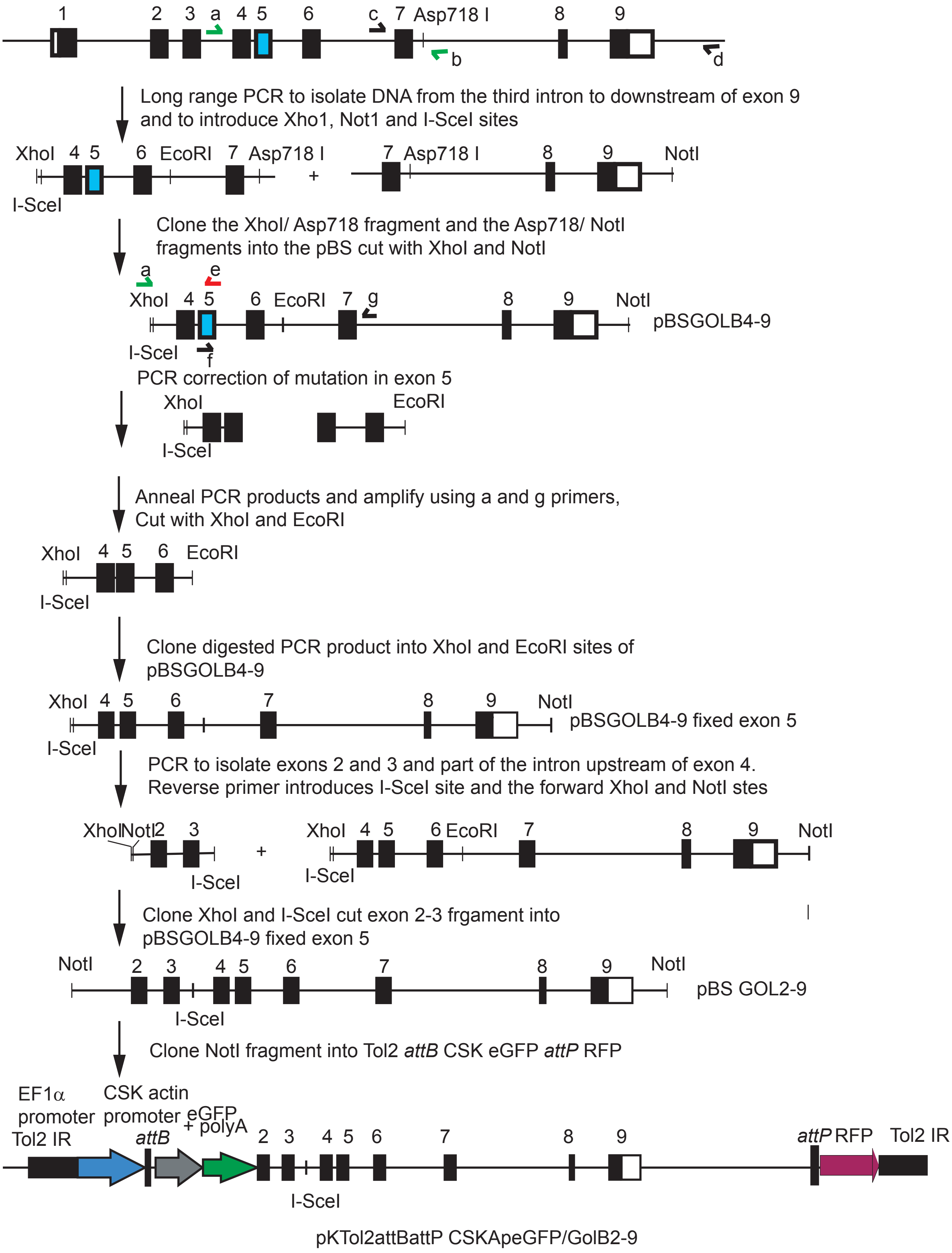

Figure 2; supplementary data Brookfield et al.

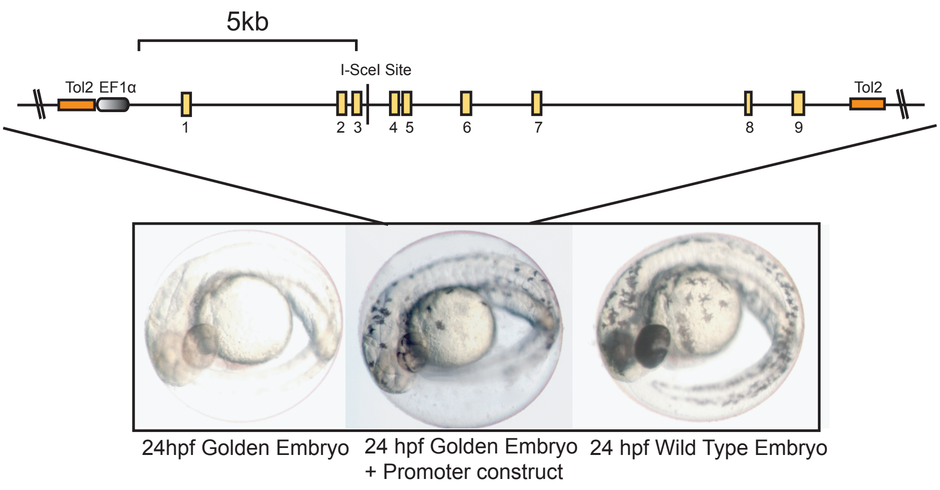

Figure 3; supplementary data Brookfield et al.

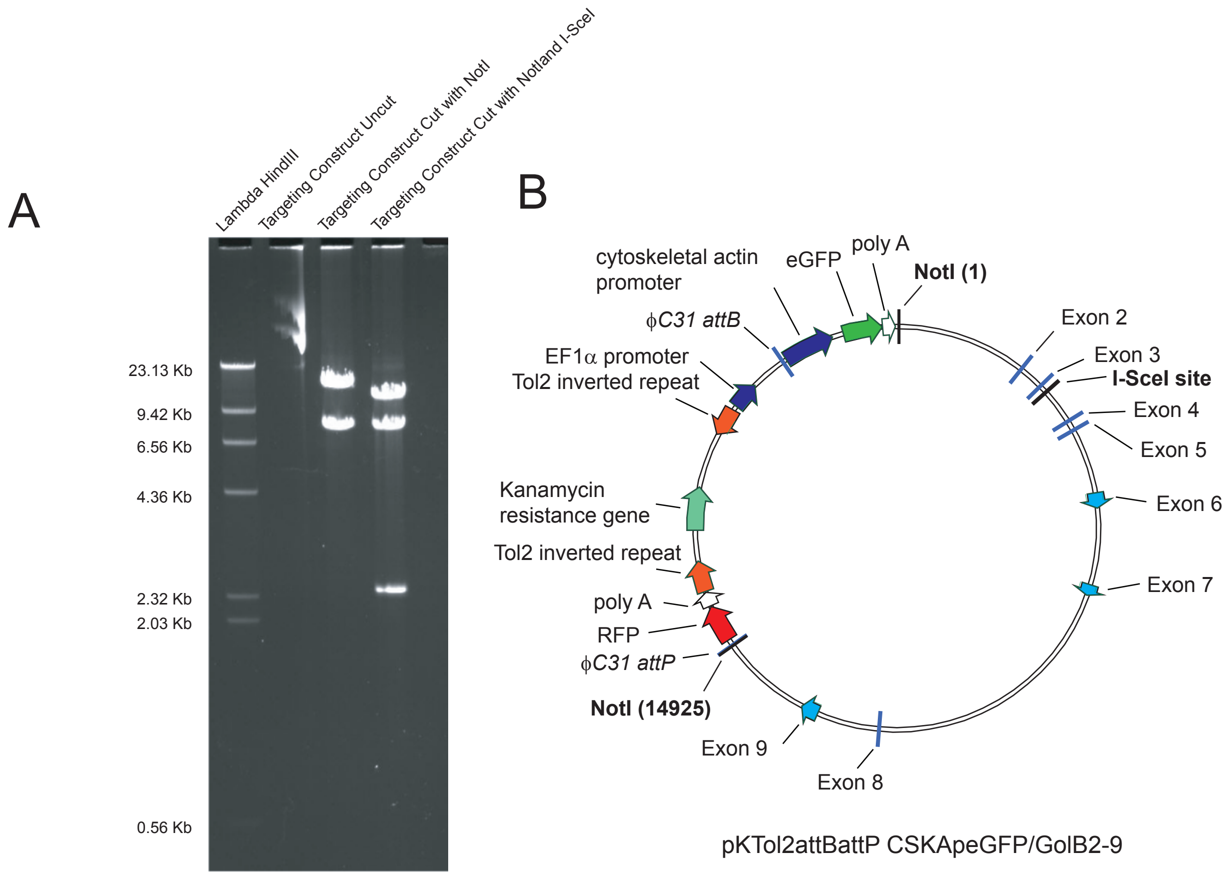

Supplement: Supplementary file 1 — Supplementary material 1 (PDF 2175 kb) [file 11248_2012_9607_MOESM1_ESM.pdf]
